# Evidence of Genetic Effects on Blood Lead Concentration

**DOI:** 10.1289/ehp.8847

**Published:** 2007-06-14

**Authors:** John B. Whitfield, Veronica Dy, Robert McQuilty, Gu Zhu, Grant W. Montgomery, Manuel A.R. Ferreira, David L. Duffy, Michael C. Neale, Bas T. Heijmans, Andrew C. Heath, Nicholas G. Martin

**Affiliations:** 1 Department of Clinical Biochemistry, Royal Prince Alfred Hospital, Sydney, Australia; 2 Genetic Epidemiology Unit, Queensland Institute of Medical Research, Brisbane, Australia; 3 Department of Psychiatry and Human Genetics, Virginia Commonwealth University, Richmond, Virginia, USA; 4 Section of Molecular Epidemiology, Leiden University Medical Center, Leiden, the Netherlands; 5 Missouri Alcoholism Research Center, Department of Psychiatry, Washington University School of Medicine, St. Louis, Missouri, USA

**Keywords:** blood lead, heritability, linkage, toxicogenetics, twin study

## Abstract

**Background:**

Lead is an environmental pollutant that causes acute and chronic toxicity. Surveys have related mean blood lead concentrations to exogenous sources, including industrial activity, use of lead-based paints, or traffic density. However, there has been little investigation of individual differences in lead absorption, distribution, or toxicity, or of genetic causes of such variation.

**Objectives:**

We assessed the genetic contribution to variation in blood lead concentration in adults and conducted a preliminary search for genes producing such variation.

**Methods:**

Erythrocyte lead concentration was measured by inductively coupled plasma mass spectrometry in venous blood samples from 2,926 Australian adult male and female twins. Mean lead concentrations were compared by place of residence, social class and education, and by the subjects’ age, sex, alcohol intake, smoking habits, iron status, and *HFE* genotype.

**Results:**

After adjustment for these covariates, there was strong evidence of genetic effects but not for shared environmental effects persisting into adult life. Linkage analysis showed suggestive evidence (logarithm of odds = 2.63, genome-wide *p* = 0.170) for a quantitative trait locus affecting blood lead values on chromosome 3 with the linkage peak close to *SLC4A7*, a gene whose product affects lead transport.

**Conclusions:**

We conclude that genetic variation plays a significant role in determining lead absorption, lead distribution within the body, or both.

Many elements and compounds present in the environment pose significant health risks to exposed individuals, and contribute to the burden of disease for society. For any exposed person the outcome may be affected by factors such as the degree of exposure, age, and genetically determined differences in uptake, elimination, or sensitivity to the toxic effects of the substance. Investigation of such genetic differences has been called toxicogenetics (Orphanides and Kimber 2003), and although most work has been done on toxic reactions to therapeutic drugs, the approach is equally applicable to the study of environmental pollutants.

There has long been concern about chronic toxic effects of the elements lead, cadmium, arsenic, and mercury, and an extensive literature exists on their associations with a range of conditions. For lead, the main community concerns are about impaired intellectual development in infancy and childhood (Canfield et al. 2003; Needleman 2004; Pocock et al. 1994; Schwartz 1994). There is also some evidence that higher lead values are associated with increased adult mortality (Lustberg and Silbergeld 2002, Schober et al. 2006), and cognitive decline in older people (Weisskopf et al. 2004).

Environmental exposure is a precondition for accumulation of these potentially toxic elements, and major changes in exposure (such as reduction in the lead content of petrol) have produced changes in indices of body burden (Luo et al. 2003; Pirkle et al. 1994; Schuhmacher et al. 1996; Wietlisbach et al. 1995). However, within the general population of a particular country or region and at a particular time, variation in exposure is probably only one of several factors influencing accumulation and body burden. There is evidence that blood lead concentrations are quite stable within people who are exposed to sources of lead only in the general environment, and that there are significant between-individual differences (Brody et al. 1994; Delves et al. 1984).

Individual differences in risk from lead may be caused by differences in behavior (such as the known effects of smoking and drinking) or by genetic differences in metabolic or transport processes. A number of attempts have been made to test for effects of genetic polymorphisms on blood or bone lead values. To date the results are mixed, with allelic associations both reported and denied for *ALAD* (aminolevulinate, delta-, dehydratase; HGNC ID 395) [all gene names, symbols, and ID numbers are from the Human Gene Nomenclature Committee (HGNC) available at http://www.gene.ucl.ac.uk/nomenclature/index.html and accessed 2 April 2007] and *VDR* (vitamin D 1,25-dihydroxyvitamin D3) receptor; HGNC ID 12679) (Kelada et al. 2001; Schwartz et al. 2000). Because of the clinical association between iron deficiency and lead toxicity, an association with iron status, which may up- or down-regulate intestinal divalent cation transporters, and specifically with *HFE* (hemochromatosis; HGNC ID 4886) genotype has been proposed (Barton et al. 1994; Wright et al. 2004). More generally, only a few studies have considered the question of familial similarity of blood lead and whether any such similarity is due to shared genes or shared environment, and once again the results are not definitive. A family-based study (Hopper and Mathews 1983; Hopper et al. 1982) showed evidence of shared environmental effects in young siblings that diminished with age, and no significant spousal correlation, whereas a twin study (Bjorkman et al. 2000) suggested additive genetic effects in women and shared environmental effects in men.

To clarify the degree of familial similarity and to determine how far genetic and shared and nonshared environmental factors contribute to variation, we have measured lead concentrations in blood samples from male and female adult twins residing in Australia. The analysis includes consideration of covariates such as sex, age, smoking, alcohol intake, and place of residence; iron status; and *HFE* genotype. We have also performed sib-pair linkage analysis using data from dizygotic (DZ) twin pairs to identify quantitative trait loci (QTLs) affecting blood lead concentration.

## Material and Methods

### Subjects

The participants in this study were twins enrolled in the Australian Twin Registry, born between 1903 and 1964. They completed a postal questionnaire in 1989, a telephone interview in 1993–1994, and provided a blood sample in 1993–1996 (Whitfield et al. 1998). Although all were twins, in some cases only one member of a twin pair provided blood. We determined zygosity from responses to questions about physical similarity and the inability of others to tell them apart, supplemented by blood group information and, for those DZ pairs participating in linkage projects (Beekman et al. 2003), genome-wide microsatellite genotyping. Participants gave written informed consent and the studies were approved by the appropriate ethics committees.

Blood was collected from 1,134 men and 2,241 women. At the same visit, their height and weight were measured and body mass index (BMI) was calculated as weight (kg)/[height (m)]^2^. Information on alcohol intake (the number of drinks in the previous week) was obtained by self-report questionnaires. Information on smoking was derived from the 1989 questionnaire, and its use for the 1993–1995 period has been validated in a previous paper (Whitfield et al. 2000b). Data on the number of years of education (in seven categories) and social class (in three categories) were extracted from self-reports in the 1989 questionnaire (Miller et al. 2001). Participants’ addresses were categorized (using their post-codes and a database on the geographic basis of Australian postcodes) into urban, suburban, or rural zones. This categorization was based on distance from the central post office in the closest major city (Whitfield et al. 2005). Because the size and population density of the cities vary, the distances used for classification also varied. For Sydney and Melbourne distances of 10 and 40 km were used to group postcodes into urban, suburban and rural categories; for Adelaide, Brisbane, Hobart, Newcastle, and Perth, 5 and 20 km; and for Canberra and Darwin no urban category was applied and a distance of 10 km divided suburban from rural.

Information on the subjects for whom erythrocyte lead results are available is summarized in Table 1. Most are of European descent, with the majority having British or Irish ancestry.

### Laboratory procedures

Blood was collected into EDTA, lithium heparin, and plain tubes. Plasma, and then the buffy coat, were removed from the anticoagulated tubes after centrifugation and the remaining red cells were stored at –20°C or below until analyzed. Serum was obtained from the plain tubes and stored at –70°C until analyzed. Erythrocytes rather than whole blood were used for analysis of lead because the original samples had been separated to maximize the amounts of plasma and buffy coat to be used for other purposes. Essentially all the lead in blood is bound to the erythrocytes, and plasma or serum lead is < 1% of blood lead (Manton et al. 2001; Schutz et al. 1996). In some cases a portion of the erythrocytes had been diluted with a sucrose solution to preserve them; these were subsequently frozen under the same conditions as the erythrocyte samples and later used for lead measurement. Before analysis, the erythrocytes were thawed at room temperature and diluted 1:20 in ammonia/EDTA solution containing rhodium as an internal standard. Lead concentrations were measured by inductively coupled plasma mass spectrometry (ICP-MS) on a Perkin-Elmer Elan 5000 (PerkinElmer Inc, Wellesley, MA, USA) or Varian UltraMass (Varian Inc, Palo Alto, CA, USA). Hemoglobin concentration was then measured on the diluted samples using the cyanmethemoglobin method.

Analytical precision was calculated from results on high and low quality control (QC) materials that were analyzed with each batch of samples. At a mean lead concentration of 0.14 μmol/L, the standard deviation was 0.018 μmol/L (coefficient of variation 13.1%) and at 1.81 μmol/L it was 0.169 μmol/L (9.4%).

Serum urate was measured by Boehringer (Mannheim, Germany) reagents and method-son a Hitachi 747 analyzer (Hatachi, Tokyo, Japan); serum ferritin, transferrin, and iron were measured using Roche Diagnostics (F. Hoffmann-La Roche Ltd, Basel, Switzerland) reagents and methods on a Hitachi 917 analyzer. *HFE* genotype was determined by polymerase chain reaction and allele-specific oligonucleotide hybridization (Whitfield et al. 2000a).

### Data analysis

A total of 2,926 individuals had erythrocyte lead measured. In addition, 571 of the individuals had lead measurements on two separate sample tubes, collected during the same venipuncture but subsequently processed and stored separately and analyzed on different occasions. Assays were carried out on 102 different days and the two QC samples were run at least once on each day.

Tests for effects of covariates and estimation of mean values by subgroups such as sex, age, alcohol intake, or smoking were initially performed using SPSS (SPSS Inc., Chicago, IL, USA). Of the 2,926 people with erythrocyte lead data, information on age and sex was available for all, but the numbers with other covariate data varied, as shown in Table 2.

Model fitting to test for effects of genetic and environmental sources of variation and multivariate analysis of covariate effects were performed using Mx (Neale 1999). Mx is a matrix algebra interpreter and numerical optimizer for statistical modeling, allowing simultaneous modeling of both fixed effects on the mean (e.g. from age, sex, and smoking status) and random effects on variances and covariances (e.g., additive genetic, shared, and non-shared environmental sources of variance). Use of Mx also overcomes potential statistical problems that can arise with analysis of data from related subjects, in this case twins. The Mx data analysis was performed on results from 2,832 people who had *HFE* genotypes available; where necessary, missing covariate data were replaced by the mean values. There were 428 monozygotic (MZ) female, 165 MZ male, 218 dizygotic (DZ) female, 90 DZ male, and 222 DZ opposite-sex pairs, and 356 female and 230 male individuals whose co-twin did not participate.

To adjust for daily assay variation, a data group was added to the Mx analysis estimating day effects from the results of the QC samples. These were equated back to daily deviations specified in the means model of MZ and DZ twins. This allows the error variation inherent in measurement of lead in the samples to be taken into account, with both QC and twin samples contributing to the maximum-likelihood estimation of and adjustment for day effects.

### Effects of covariates

First, an analysis including the data from duplicate measurements was undertaken. Adjustments were made for sample hemoglobin concentration, effects of day-to-day analytical variation (incorporating QC data and batch effects assessed from the mean for all samples analysed within a batch), sex, and age. At each step, the improvement in goodness-of-fit was assessed by the likelihood-ratio chi-square test as each of the variables was added. As anticipated, significant effects were found for day-to-day analytical variation, sample hemoglobin concentration, age, and sex (all *p* < 10^–6^). The test–retest repeatability after allowing for all these was *r* = 0.79, with 95% confidence interval (CI) 0.75–0.82.

Second, we tested for the effects of geographic, social, and metabolic characteristics hypothesized to affect lead results. These were sex, age, drinking habits, smoking status, residential location (categorized as urban, suburban or rural, as defined above), social class (in three categories), educational level (seven categories), BMI, serum uric acid, serum ferritin and transferrin saturation, and *HFE* CY and HD genotypes. To simplify the analysis, the first or only lead value for each person tested was used. Univariate analysis using SPSS was supplemented with Mx analysis to assess the independent effects of these covariates. In this, the initial or baseline model contained all the covariates; a series of submodels were then fitted in which one and only one of the covariates was removed and the change in goodness-of-fit calculated to determine the significance of their independent contribution.

### Sources of variation

As the simultaneous adjustment for all the covariate effects was computationally intensive, we took advantage of a new feature of Mx 1.57 (developed for this application) and saved residuals from the model containing all the covariates. These residuals were used to fit models of genetic and environmental sources of variation to the within- and between-pair covariances by zygosity group, as previously described (Whitfield et al. 2000a) and also for the linkage analysis. Models were initially tested with additive genetic (A), non-shared environmental (E), and either dominant genetic (D) or shared environmental (C) sources of variation. The ACE or ADE models were then compared with models containing only A and E, only C and E, or E alone.

### Linkage analysis

We performed a genome scan of erythrocyte lead levels on 414 of the DZ twin pairs. DNA was extracted from blood or buccal swabs according to standard procedures (Miller et al. 1988). Genotyping data were collected from at least one of four genome scans that had previously been performed for other projects by the Mammalian Genotyping Service (Marshfield, WI, USA; Leiden University Medical Centre, the Netherlands) (Beekman et al. 2003); Sequana Therapeutics Inc. (La Jolla, CA, USA) and Gemini plc (Cambridge, UK). Familial relationships were verified using GRR (graphical representation of relationship errors; Abecasis et al. 2001). After errors were resolved, Mendelian segregation inconsistencies were identified and removed with SIB-PAIR computer software, version 0.99.9 (Duffy 2005a). Genotypes associated with unlikely recombination events were subsequently flagged and wiped with MERLIN version 0.10.1 (Abecasis et al. 2002). The marker genetic positions were interpolated via locally weighted linear regression from the National Center for Biotechnology Information Build 34.3 physical map positions (National Center for Biotechnology Information 2007) and the published “Rutger’s” (Kong et al. 2004) genetic map (Duffy 2005b). Genetic positions are expressed in Kosambi centiMorgans (cM). Of the 414 available sib-pairs, 388 (94%) were genotyped at 300–1,544 markers (mean 730) and 28 (6%) at less than 300 markers. Linkage analyses with or without this latter group of sib-pairs provided similar results, so we present only the analysis including all families. The mean intermarker distance for sib-pairs with 300 or more markers in common, estimated for each sib-pair and then averaged across the 388 pairs, was 5.7 cM (SD = 2.9; range, 1.8–49.4). The average multipoint entropy information content (Kruglyak et al. 1996) was 0.54, computed with MERLIN at the marker closest to the middle of the chromosome and averaged over the 22 autosomes. The average heterozygosity for the autosomal markers was 75%. Finally, sibling IBD (identity by descent) sharing was estimated via multipoint methods on a 5-cM grid and maximum likelihood univariate variance components linkage analysis performed as implemented in MERLIN, after adjustment for all the tested covariates. At every 5 cM, a 1-degree-of-freedom (df) logarithm of odds (LOD) score was computed, which is distributed as a 50:50 mixture of a point probability mass at 0 and a χ_1_^2^, being equivalent to the original parametric LOD score proposed by Morton (1955).

### Empirical genome-wide thresholds

Trait-specific empirical genome-wide suggestive and significant thresholds were calculated using 1,000 gene-dropping simulations as described by Abecasis et al. (2004). For each simulation, we used MERLIN to generate a new data set with the original phenotypes but with new genotypes simulated under the null hypothesis of no linkage for all autosomal markers, retaining the same allele frequencies, marker spacing, and missing data pattern. Multipoint IBDs for each simulation replicate were then computed and linkage analysis performed as described above. We recorded the highest peak observed for each chromosome and counted the number of chromosomes exhibiting an LOD score equal to or greater than a given threshold *p*. The empirical genome-wide thresholds for suggestive or significant linkage (Lander and Kruglyak 1995) were defined as the thresholds *p* for which we observed on average 1 or 0.05 peaks per simulation with a LOD score ≥ *p*, respectively.

## Results

### Effects of covariates

The mean value for lead concentration in all the samples before adjustment for covariates was 0.388 μmol/L, and the mean hemoglobin concentration was 235 g/L. The erythrocyte lead concentrations, extrapolated to whole blood with an average hemoglobin concentration of 140 g/L, imply mean lead concentrations in whole blood of 0.265 μmol/L (SD 0.165) for men, and 0.213 μmol/L (SD 0.125) for women. The results of the significance tests for the demographic and metabolic factors tested for their associations with the erythrocyte lead values are summarized in Table 2. Of the variables hypothesized to affect or co-vary with lead concentrations, the strongest univariate associations were for age, alcohol intake, sex, serum uric acid concentration, and smoking status. The geographic and social indicators, place of residence, and social class, had variable or nonsignificant effects, but educational level had significant and consistent effects in both men and women.

Multivariate analysis (Table 3) showed that sex, age, drinking, and smoking all had independent effects. Educational level showed only marginally significant effects in multivariate analysis, and both residential location and social class were nonsignificant. BMI and serum ferritin had significant but small effects. Other factors associated with iron status, the serum transferrin saturation and *HFE* genotypes, had no independent effect. A highly significant and independent association between erythrocyte lead and plasma uric acid was confirmed. Among the results that were found to be statistically significant, men had higher erythrocyte lead values than women. Mean lead values rose with age, with smoking, and with increasing alcohol intake. Both ferritin and urate values were positively correlated with erythrocyte lead concentration.

### Sources of variation

After adjustment for all the covariate effects, the correlations between residuals for members of twin pairs, by zygosity were r_MZF_ 0.43, r_MZM_ 0.46, r_DZF_ 0.24 r_DZM_ 0.21, and r_DZFM_ 0.18. There was no evidence of differences in correlation between men and women, and pooled zygosity correlations were r_MZ_ 0.43 (95% CI, 0.37–0.50), r_DZ_ 0.22 (95% CI, 0.14–0.30). These correlations were affected very little by dropping covariates from the model (Table 3) except in the case of age. Because members of a twin pair are of the same age regardless of zygosity, and age had a substantial effect on lead values, dropping the age effect produced an increase in within-pair correlations in both MZ and DZ pairs.

Tests of the sources of residual variation in erythrocyte lead are shown in Table 4. First, there was clear evidence of familial similarity because the model containing only nonshared environmental effects (E) was very strongly rejected when compared with any of the others. Second, the source of this family resemblance was genetic rather than due to long-term effects of the shared family environment. The AE model, containing additive genetic and non-shared environmental sources of variation, gave a much better fit to the data than the CE model containing shared and nonshared environmental sources of variation (*p* = 0.00003). When the AE model was expanded to contain either C (shared environment) or D (dominant genetic effects) both the C and D effects were estimated at zero. However, the upper 95% CI for the estimate of C in the ACE model is 16%, so we cannot exclude some persisting effects of the shared environment.

### Linkage analysis

**Figure 1:**
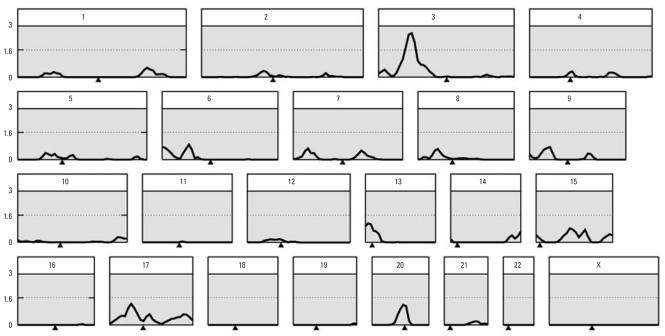
Results of linkage analysis for erythrocyte lead (residuals after adjusting for covariates). Each panel represents one chromosome; the *x*-axis shows genetic distance from the p-terminal end, and centromeres are indicated by filled triangles below this axis. The *y*-axis shows LOD scores, and the lines at 3.0 and 1.6 on the γ-axis represent the empirical “significant” and “suggestive” values for genome-wide significance as determined by simulation.

**Figure 2:**
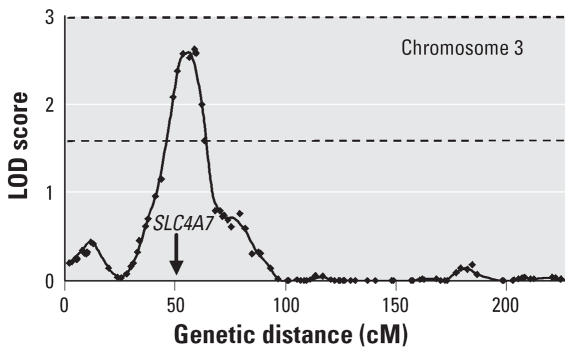
Linkage results for chromosome 3. The *x*-axis shows genetic distance from the p-terminal end and the *y*-axis shows LOD scores, and the lines at 3.0 and 1.6 on the *y*-axis represent the empirical “significant” and “suggestive” values for genome-wide significance as determined by simulation. Arrow indicates the position of *SLC4A7*.

In the linkage analysis, the highest LOD score was found on chromosome 3 (2.63 at D3S1619, or 2.59 at 57.2 cM on the 5 cM grid). The empirical significance threshold was estimated by simulation, and it was found that a value ≥ 2.63 was observed in only 170 of 1,000 simulations (i.e., genome-wide *p* = 0.17). Three other regions showed peak LOD scores > 1.0. These were on chromosomes 13 (1.17 at D13S787), 17 (1.27 at ATA78D02), and 20 (1.42 at D20S107). The empirical genome-wide threshold for suggestive linkage was 1.59 and for significant linkage 3.00. Thus only the chromosome 3 peak exceeds the suggested threshold. The overall linkage results, and an enlarged plot for chromosome 3, are shown in Figures 1 and 2.

## Discussion

### Sources of variation in blood lead

We set out to investigate the causes of variation in blood lead concentrations in the general population of Australia, using samples that we had collected from a large series of adult twins living throughout the country in the early 1990s. One expectation was that geographic differences, particularly those between innercity and rural environments, would be a significant source of variation. Other influences shared within families, such as social class or perhaps educational levels, might also be significant. There was indeed a strong familial component to lead variation, but this proved to be genetic in origin. This leads to a different perspective on the true sources of variation within this population.

One major finding is the significant heritability of blood lead concentrations. The data clearly indicate the existence of genetic contributions to variation in blood lead, with MZ pair correlations twice DZ pair correlations and no evidence of differences in correlation between male and female pairs or between same-sex and opposite-sex DZ pairs. These correlations were little changed when covariate effects were dropped from the model, remaining twice as great in MZ as in DZ pairs, as shown in Table 3. Estimated heritability was just over 40% (Table 4). There is little prior information on variation in blood lead within a twin- or family-study design. The conclusions of two previous studies differ from each other and also from our results. A comparison of blood lead levels within families (Hopper and Mathews 1983) led to the conclusion that the shared environment was important in children because high correlations were observed for young siblings living together, but these correlations diminished with age. The only previous twin study (Bjorkman et al. 2000) found evidence for genetic effects on blood lead in women but not in men; in the men the high within-DZ-pair correlation suggested persisting effects of shared environment, even though the subjects were 49–86 years of age and had presumably lived apart for 30–70 years.

Although some shared environmental effects could not be excluded in our data (up to 16% of variance; Table 4), the maximum-likelihood estimate was zero. This is contrary to the earlier Australian finding of the importance of familial environment (Hopper et al. 1982). However these results may be reconciled if the relative importance of genetic and environmental variation has changed over time, particularly as the amount of lead entering the environment from petrol has decreased and environmental differences have been reduced. In Switzerland at least, there was a convergence of city and country blood lead values as unleaded petrol came into use (Wietlisbach et al. 1995).

### QTLs for blood lead

The second major finding arises from the linkage results. Despite the limited power of linkage analysis to detect QTLs with only 416 DZ twin pairs, we found a region of suggestive linkage on chromosome 3 with an LOD score of 2.65 at D3S2396 (Figure 2). This marker is located 54.06 cM or 29.76 Mb from the p-terminal end of the chromosome. Examination of a list of genes within the 1-LOD confidence interval of this peak, from 24 to 39 Mb, shows 62 genes or putative genes. Many are unidentified and nearly all lack obvious relevance for blood lead. However one, *SLC4A7* (solute carrier family 4, sodium bicarbonate cotransporter, member 7; HGNC ID 11033), codes for a bicarbonate transporter (Pushkin et al. 1999). Movement of lead into erythrocytes (and possibly other tissues) has been shown to depend on bicarbonate transport, being blocked by inhibitors of anion transport (Bannon et al. 2000; Simons 1986). This leads to the hypothesis that polymorphic variation in *SLC4A7*, in coding or regulatory sequences, produces the genetic variation in erythrocyte lead concentration that we observed. Although we recognize that other genes may be the source of the chromosome 3 linkage peak through mechanisms that are not immediately apparent, *SLC4A7* is a good candidate and demands further investigation.

Another region of interest is on chromosome 20. The peak LOD score of 1.42 does not exceed the suggestive threshold but coincides with the location of another gene, which may affect anion transport and therefore the accumulation of lead within cells. This gene, *EPB41L1* (erythrocyte membrane protein band 4.1-like-1, HGNC ID 3378), codes for a member of the protein 4.1 family. These proteins bind to ion transport proteins and provide a physical connection between the cytoskeleton and the cell membrane. It is believed that such binding plays a role in regulating the activity of ion transport proteins (Denker and Barber 2002). Although the chromosome 20 linkage peak is not strong, this possible physiologic connection with the more substantial chromosome 3 peak is of interest.

Conversely, we can compare our linkage results to the locations of candidate genes. These include *ALAD* (chromosome 9, 111.5 Mb), *VDR* (chromosome 12, 46.5 Mb), *HFE* (chromosome 6, 26.2 Mb), metallothioneins (*MT1B* metallothionein 1B, HGNC ID 7394; *MT1E* metallothionein 1E, HGNC ID 7397; *MT1G* metallothionein 1G, HGNC ID 7399; *MT1M* metallothionein 1M, HGNC ID 14276; *MT2A* metallothionein 2A, HGNC ID 7406; *MT3* metallothionein 3, HGNC ID 7408; *MT4* metallothionein 4, HGNC ID 18705; all chromosomes 16, 56.4 Mb), divalent metal transporter (*SLC11A2*; solute carrier family 11 (proton-coupled divalent metal ion transporters), member 2 , HGNC ID 10908; chromosome 12, 49.7 Mb), hepcidin (*HAMP*; hepcidin antimicrobial peptide, HGNC ID 15598; chromosome 19, 40.5 Mb), and iron regulatory proteins (*ACO1*; aconitase 1, soluble, HGNC ID 117; chromosome 9, 32.4 Mb and *IREB2*, iron-responsive element binding protein 2, HGNC ID 6115; chromosome 15, 76.4 Mb). Although *HFE* is close to a minor peak (LOD score 0.89 at D6S2439, at 24.4 Mb, less than 2 Mb from *HFE*), none of the candidate genes are situated in genomic regions with LOD scores above 1.0 in our linkage analysis. This does not rule out their involvement at levels below our threshold for detection by linkage, which has less power than allelic association to detect small effects. It is also possible that some effects are confined to a comparatively rare genotype, such as the YY genotype for *HFE*, and the number of DZ pairs with such genotypes is insufficient to detect linkage.

### Effects of measured covariates

Third, we have tested for effects of a number of covariates that have been reported to affect blood lead levels in other population-based studies. The significant effects of age, sex, smoking, and alcohol use are similar to those previously reported (Brody et al. 1994; Shaper et al. 1982; Weyermann and Brenner 1997). Mean values are consistent with those reported elsewhere; our mean value for men is similar to that reported for non-Hispanic white men in the United States in 1988–1991 (0.18–0.23 μmol/L, depending on age group), but our mean for women is higher than for U.S. non-Hispanic white women (0.08–0.17 μmol/L) (Brody et al. 1994). Comparable adult Australian data are not available, but a survey of children in Australia in 1995 found a mean value of 0.28 μmol/L (Donovan 1996). It is not clear why the difference between adult male and female lead concentrations should be less in Australia than in the United States.

Education showed a trend for decreasing lead values with increasing years of education. This association has been described in other studies. In the NHANES III study (Brody et al. 1994) a difference was found between high school graduates and others, and our data (Table 2) suggest the type of post-school training or education undertaken makes little further difference. Negative results in the multivariate analysis may be because of the nonlinear relationship between the educational grouping and lead levels or association between education and other predictor variables in the analysis. Other measures of environmental exposure (residential location) and socioeconomic status (self-reported social class) did not show independent effects on lead concentration, although minor effects of residential location were seen for women in the univariate analysis. U.S. data (Brody et al. 1994) do suggest that place of residence affects blood lead, but the contrasted areas (central city with population > 1 million, < 1 million, or other locations) are not easily comparable with our classifications and the nature of the central-city environment differs in Australia. To some extent, education, residence, and social class will be similar within twin pairs; shared environmental effects on residential location persist into adult life and genetic effects displace them in the older participants in this study (Whitfield et al. 2005). However, because the measures of environmental exposure did not show significant effects on lead levels, they are unlikely to have affected the comparison of MZ and DZ pair similarities.

The association with ferritin in the multivariate analysis was in a positive direction; higher lead values were found in those with higher ferritin. However, the univariate analysis failed to show a clear trend across the ferritin categories, in either men or women. We found no significant associations between lead and transferrin saturation. For *HFE* genotype, results were nonsignificant, but it may be noted that in both men and women the minor allele homozygotes (YY or DD) had the lowest mean lead levels. Lower bone lead values in carriers of variant *HFE* alleles have been reported by others (Wright et al. 2004).

The magnitude of the positive association between uric acid and lead was unexpected, although some previous reports have shown associations in the general population (Lin et al. 2002; Shadick et al. 2000). It appears that lead exposure reduces the renal excretion of urate because chelation therapy increases urate clearance (Lin et al. 2002).

## Limitations and Conclusions

The conclusions of this study, although clear, are subject to a number of limitations. First, the samples were collected about 10 years ago, and environmental lead has decreased further since that time. This environmental change will have reduced the mean values for blood (or erythrocyte) lead in the population and may have reduced variability. Second, our study was on adults from the general population and cannot directly illuminate causes of variation in vulnerable groups such as young children or more highly exposed groups such as lead workers. Because much of the lead exposure of infants is from ingestion of lead paint residues in soil surrounding older houses, the balance between environmental and genetic causes of variation may be different in children than in adults. Third, we only measured lead in blood, so the factors described may affect lead absorption and body burden, or only its distribution within the body. In the latter case some people could have high blood lead levels but an acceptable total body content or vice versa. Distinguishing between these possibilities would require measurement of lead in bones or perhaps in deciduous teeth. Finally, it is sometimes claimed that MZ twin pairs are more similar in their environments than DZ pairs. In studies of biochemical characteristics in adult twins, this is unlikely to be important; even if MZ pairs did share more of their environmental exposure to lead up to 16 or 18 years of age, it would be remarkable if this produced a greater similarity in blood concentrations some 30 years later.

Overall, our results emphasize the significance of genetic factors in determining individual differences in blood lead concentration, at least in adults. They also suggest a role for an ion transporter gene whose product mediates lead transport into cells. However, we should not infer from this that environmental exposure is unimportant or that efforts to reduce exposure to lead have low priority or lack efficacy. To modify a well-known metaphor, a falling tide will lower all boats.

## Figures and Tables

**Table 1:** Descriptive information for participants with erythrocyte lead data.

Grouping	Proportion of subjects (%) or mean ± SD
Sex	
Male	34
Female	66
Age (years)	46.0 ± 11.8
Drinks in previous week	
None	34
1–7	39
8 –14	15
15–21	7
22–28	2.5
> 28	2.5
Smoking	
Smokers	20
Nonsmokers	80
Residence	
Urban	12
Suburban	44
Rural	44
Education	
< 7 years	1
8–10 years	26
11–12 years	23
Apprenticeship, diploma	18
Technical/teachers’ college qualification	14
University, first degree	11
University, postgraduate training	7
Social class	
Working	32
Middle	52
Upper	15
BMI	
< 25	54
25–30	34
> 30	12
Uric acid	
Male (μmol/L)	0.386 ± 0.077
Female (μmol/L)	0.285 ± 0.073
Ferritin (log_10_ μg/L)	
Male	2.253 ± 0.357
Female	1.812 ± 0.421
Transferrin saturation	
Male	27.2 ± 9.2
Female	24.9 ± 10.8
*HFE* H63D	
HH	74
HD	24
DD	2
*HFE* C282Y	
CC	86
CY	13
YY	0.6

**Table 2:** Effects of sociodemographic, substance use and biological factors on erythrocyte lead (univariate analyses, adjusted for sample hemoglobin concentration).

	Female			Male		
Grouping	Mean	SE	*n*	Mean	SE	*n*
All participants	0.358	0.005	1,925	0.445	0.006	1,001
	*p* < 0.01					
Age (years)						
Up to 35	0.290	0.009	373	0.421	0.016	207
36–45	0.312	0.006	696	0.431	0.011	415
46–55	0.384	0.008	445	0.491	0.016	213
56–65	0.436	0.011	227	0.506	0.025	83
> 65	0.455	0.012	184	0.512	0.025	83
	*p* < 0.001			*p* < 0.001		
Drinks						
None	0.341	0.006	761	0.426	0.015	219
1–7	0.340	0.006	805	0.424	0.012	339
Up to 14	0.392	0.011	235	0.471	0.016	201
15–21	0.382	0.020	75	0.492	0.020	132
22–28	0.540	0.032	29	0.519	0.034	44
> 28	0.539	0.058	9	0.545	0.029	63
	*p* < 0.001			*p* < 0.001		
Smoker						
No	0.346	0.005	1,469	0.434	0.008	748
Yes	0.372	0.009	376	0.518	0.016	190
	*p* < 0.05			*p* < 0.001		
Residence						
Urban	0.390	0.013	198	0.453	0.021	125
Suburban	0.347	0.006	772	0.449	0.011	441
Rural	0.344	0.006	821	0.468	0.012	374
	*p* < 0.01			NS		
Education						
< 7 years	0.442	0.037	22	0.477	0.073	9
8–10 years	0.382	0.007	581	0.530	0.018	146
11–12 years	0.333	0.008	463	0.433	0.017	168
Apprenticeship, diploma	0.349	0.010	279	0.478	0.015	209
Technical/teachers’ college qualification	0.329	0.011	262	0.426	0.019	126
University first degree	0.333	0.015	142	0.412	0.017	175
University postgraduate training	0.321	0.018	96	0.402	0.021	106
	*p* < 0.001			*p* < 0.001		
Social class						
Working	0.347	0.007	612	0.483	0.013	315
Middle	0.350	0.006	1,011	0.429	0.010	508
Upper	0.373	0.011	285	0.470	0.018	157
	NS			*p* < 0.01		
BMI						
< 25	0.348	0.005	1,124	0.442	0.011	434
25–30	0.366	0.008	523	0.465	0.011	452
> 30	0.345	0.011	253	0.459	0.023	101
	NS			NS		
Urate						
Q1	0.331	0.010	260	0.397	0.016	173
Q2	0.313	0.009	318	0.402	0.017	162
Q3	0.351	0.011	250	0.424	0.017	149
Q4	0.339	0.010	256	0.491	0.017	157
Q5	0.376	0.010	284	0.481	0.017	160
	*p* < 0.001			*p* < 0.001		
Ferritin						
Q1	0.367	0.008	367	0.419	0.016	188
Q2	0.337	0.008	364	0.466	0.016	188
Q3	0.356	0.009	360	0.490	0.016	191
Q4	0.348	0.010	367	0.443	0.016	187
Q5	0.333	0.014	363	0.423	0.016	190
	*p* < 0.05			*p* < 0.01		
Tf sat						
Q1	0.353	0.009	432	0.418	0.021	116
Q2	0.344	0.009	370	0.409	0.017	182
Q3	0.358	0.009	346	0.460	0.016	212
Q4	0.354	0.010	335	0.483	0.015	216
Q5	0.330	0.010	338	0.451	0.015	218
	NS			*p* < 0.05		
*HFE* CY						
CC	0.351	0.004	1,594	0.457	0.008	848
CY	0.379	0.011	259	0.462	0.021	116
YY	0.296	0.046	15	0.398	0.133	3
	*p* < 0.05			NS		
*HFE* HD						
HH	0.354	0.005	1,402	0.463	0.009	684
HD	0.355	0.009	417	0.452	0.014	258
DD	0.335	0.028	42	0.343	0.051	21
	NS			NS		

NS, not significant; Q, quintile; TF, transferrin saturation.

**Table 3:** Effects of sociodemographic, substance use, and biological factors on erythrocyte lead (multivariate analysis).

Models	[Table-fn tfn2-ehp0115-001224]–2LL	df	Δχ^2^	Δdf	*p*-Value	Percent of variance	r_MZ_	r_DZ_
Baseline	[Table-fn tfn2-ehp0115-001224]–4007.86	3,138	—	—	—	—	0.425	0.213
Sex	[Table-fn tfn2-ehp0115-001224]–3956.39	3,139	51.46	1	< 0.0001	3.2	0.430	0.199
Age and age^2^	[Table-fn tfn2-ehp0115-001224]–3772.10	3,140	235.76	2	< 0.0001	18.7	0.476	0.275
Drinking	[Table-fn tfn2-ehp0115-001224]–3929.50	3,139	78.36	1	< 0.0001	5.7	0.428	0.221
Smoking	[Table-fn tfn2-ehp0115-001224]–3971.73	3,139	36.12	1	< 0.0001	2.6	0.425	0.214
Residence	[Table-fn tfn2-ehp0115-001224]–4005.63	3,139	2.23	1	0.136	< 1	0.426	0.213
Education	[Table-fn tfn2-ehp0115-001224]–4002.30	3,139	5.56	1	0.018	< 1	0.427	0.211
Social class	[Table-fn tfn2-ehp0115-001224]–4007.84	3,139	0.02	1	0.888	< 1	0.425	0.213
BMI	[Table-fn tfn2-ehp0115-001224]–3998.34	3,139	9.52	1	0.002	< 1	0.421	0.212
Uric acid	[Table-fn tfn2-ehp0115-001224]–3961.75	3,139	46.10	1	< 0.0001	3.2	0.434	0.204
Ferritin	[Table-fn tfn2-ehp0115-001224]–3998.35	3,139	9.50	1	0.002	< 1	0.427	0.207
Saturation	[Table-fn tfn2-ehp0115-001224]–4005.52	3,139	2.34	1	0.126	< 1	0.424	0.215
*HFE* H63D	[Table-fn tfn2-ehp0115-001224]–4006.70	3,139	1.16	1	0.282	< 1	0.426	0.215
*HFE* C282Y	[Table-fn tfn2-ehp0115-001224]–4007.05	3,139	0.80	1	0.371	< 1	0.426	0.214

–2LL, –2 times the log likelihood. The baseline model includes adjustment for effects of all covariates listed, plus sample haemoglobin concentration and day effects. Note that the number of degrees of freedom in the second column reflects both the number of subjects tested and the number of quality control sample results in the QC data group. Each of the listed covariates in turn was dropped from the full model and then replaced, and the change in goodness of fit (Δχ^2^), and the change in total variance were estimated. MZ and DZ pair correlations are shown for the baseline model including all covariates, and after dropping each.

**Table 4:** Tests of alternative models of sources of variation in erythrocyte lead after adjustment for covariates.

							Proportions of variance			
Model	–2LL	df	Compared against	Δχ^2^	Δdf	*p*-Value	A% (95% CI)	C% (95% CI)	D% (95% CI)	E% (95% CI)
ACE	–2,019.93	2,828					42.5 (23–48)	0.0 (0–16)	—	57.5 (52–64)
ADE	–2,019.93	2,828					42.5 (10–48)	—	0.0 (0 to 34)	57.5 (51–64)
AE	–2,019.93	2,829	ACE	0	1	1.00	42.5 (36–48)	—	—	57.5 (52–64)
CE	–2,002.31	2,829	ACE	17.62	1	0.00003	—	32.0 (27–37)	—	68.0 (63–73)
E	–1,880.84	2,830	AE	139.09	1	< 10^–6^	—	—	—	100.0

Abbreviations: LL, log likelihood; df, degrees of freedom. Comparison between models was based on the change in goodness-of-fit between the model and the data (Δχ^2^) as potential sources of variation were removed. Sources of variation: A, effects due to additive actions of genes; D, effects due to actions of dominant genes; C, effects due to common environment shared by members of a twin pair; and E, effects due to unique environment not shared by members of a twin pair.
